# Study on Association Between Gut Microbiota, Serum Metabolism and Gestational Diabetes Mellitus Based on Metagenomic and Metabolomics Analysis

**DOI:** 10.3390/nu18030381

**Published:** 2026-01-23

**Authors:** Wenduo Yu, Kun Tang, Rongjing An, Sujuan Ma, Hongzhuan Tan, Mengshi Chen

**Affiliations:** 1 Department of Epidemiology and Health Statistics, Xiangya School of Public Health, Central South University, No. 172 Tongzipo Road, Yuelu District, Changsha 410013, China; windowsy@csu.edu.cn (W.Y.); ktang@csu.edu.cn (K.T.); msj1008@163.com (S.M.); tanhz@csu.edu.cn (H.T.); 2 Chaoyang District Center for Diseases Prevention and Control of Beijing, Beijing 100020, China; an1503416@163.com

**Keywords:** gestational diabetes mellitus (GDM), gut microbiota, metabolomics, first trimester, metagenomics, plasma metabolites

## Abstract

**Background/Objectives:** This study aimed to explore the association between maternal gut microbiota and metabolic profiles in the first trimester and the subsequent risk of gestational diabetes mellitus (GDM), as well as to characterize association patterns linking gut microbiota, serum metabolites, and metabolic traits. **Methods:** A nested case–control study was conducted among women with GDM (*n* = 47) and those without GDM (*n* = 94). Metagenomic sequencing was applied to analyze fecal microbiota, and liquid chromatography–mass spectrometry (LC–MS) was used for non-targeted plasma metabolomics. Differential microbiota and metabolites between groups were identified, and correlation analyses were conducted to assess their associations with clinical indicators. **Results:** Women who later developed GDM showed lower alpha diversity and higher beta diversity. Eleven differential species were identified, with *Collinsella aerofaciens* and *Clostridium bartlettii* enriched in GDM, while nine species such as *Alistipes putredinis* and *Bacteroidales bacterium ph8* were enriched in controls. Sixty-four plasma metabolites differed between groups, including increased glycerol-3-phosphate, aromatic amino acids, and glycerophosphocholine, and decreased cysteine, tryptophan, niacinamide, and stearic acid. Correlation analyses revealed significant relationships between *Alistipes putredinis*, *Eubacterium eligens*, and *Bacteroidales bacterium ph8* with metabolic and clinical indicators (e.g., TG, TC, LDL). **Conclusions:** In this nested case–control study, women who later developed GDM exhibited reduced gut microbial diversity and altered metabolic profiles during the first trimester of pregnancy. Several microbial taxa and microbiota–metabolite associations were observed in relation to subsequent GDM status, highlighting early-pregnancy microbial and metabolic features that may be relevant to GDM-related metabolic changes.

## 1. Introduction

In recent decades, the incidence of gestational diabetes mellitus (GDM) has continuously increased [1], seriously affecting the health of both mothers and infants [2,3,4].

It has been well confirmed that the gut microbiota can produce a variety of compounds that modulate the activity of the distal organs and also play a role in the development of GDM [5,6]. Alteration in the diversity of bacterial species and the abundance of specific bacterial taxa have been observed in GDM women [7,8,9,10,11,12,13,14,15,16]. The main dysbiosis of gut microbiota in pregnant women with GDM is the abundance of *Firmicutes* (or Bacillota) and *Bacteroidetes* (or Bacteroidota) or changes in F/B ratio [8,9,10]; an increase in phyla *Firmicutes* [11,12,13,14] or F/B ratio [14,15,16,17] was observed in women with GDM. However, a decrease in phyla *Firmicutes*, or reduced F/B ratio (mainly reduced class Clostridia), in the T2D patients was also observed [14,15,16,17,18,19].

Alternated composition of other phyla, such as *Proteobacteria* [17], *Pseudomonadota* [20], *Actinobacteria* [14,17], *Proteobacteria* [17], *Verrucomicrobia* [16,17], and *Fusobacteria* [17], have been observed to be associated with GDM. Genera such as *Actinomyces* [16,21], *Bacteroides* [14,16,22,23,24,25], *Blautia* [9,12,26,27], *Streptococcus* [7], and *Prevotella* [11,16] were predominantly enriched in the gut microbiome of women with GDM, while genera like *Bifidobacterium* [24,26,28] were relatively depleted. Numerous studies have identified significant differences in the abundance of many species. Among them, *Bacteroides massiliensis* [29] and *Parabacteroides distasonis* [6] were enriched in patients with GDM. Additionally, *Clostridium_spiroforme*, *Eubacterium_dolichum*, and *Ruminococcus_gnavus* were positively correlated with fasting blood glucose (FBG) levels [10].

*Anaerostipes hadrus* were associated with an impaired glucose tolerance [22]. Several species depleted in patients with GDM were observed, including *Ruminococcus* and *Alistipes*, *putredinis*, *Bifidobacterium dentium* [7,13,16,29], and *Pyramidobacter piscolens* [10], compared with normal pregnant women.

During pregnancy, particularly in the mid- and late-term, women experience a series of metabolic changes, such as increased adiposity and decrease in insulin sensitivity, which leads to an increased risk of diabetes in women in the third trimester. Results of a recent meta-analysis revealed that 67 metabolites differed between patients with GDM and controls. Among them, 25 metabolites were significantly associated with GDM risk, reflecting disruptions in multiple metabolic pathways, including amino acid, lipid, carbohydrate, and energy metabolism. Specifically, elevated levels of isoleucine, leucine, C16:0, glucose, pyruvate, lactate and other metabolites were linked to an increased risk of GDM, whereas higher concentrations of glutamine, histidine, and related metabolites were associated with a reduced risk [30]. Ye et al. [31] reported that, compared with the control group, patients with GDM had an increased abundance of 2-hydroxybutyric acid and L-alpha-aminobutyric acid, but a decrease in methionine sulfoxide, allantoin, and dopamine and dopaminergic synapse. Previous studies found that disturbances in phenylalanine and tyrosine metabolism may serve as potential biomarkers for GDM [29,30,32,33]. Sun et al. [29] further demonstrated that alterations in gut microbiome composition and microbial metabolic pathways related to carbohydrate and lipid metabolism were associated with GDM during pregnancy.

Research has found that women with GDM have a higher abundance of carbohydrate metabolism-related gut microbiota compared with control women [24], such as *Ruminococcaceae*, *Parabacteroides distasonis*, and *Prevotella*, which are believed to be associated with metabolic pathways for carbohydrate metabolism and insulin signaling [21]. In women with GDM combined with hyperlipidemia, the relative abundances of *Streptococcus*, *Faecalibacterium*, *Prevotella*, *Haemophilus*, and *Actinomyces* were significantly increased, and these bacterial abundances were positively correlated with elevated total cholesterol levels [16]. Additionally, patients with GDM with obesity exhibited pronounced intestinal microbiota dysbiosis [34]. These findings suggest that gut microbiota dysbiosis may be associated with abnormal carbohydrate and lipid metabolism, and could play a role in the pathogenesis of GDM.

More and more observational studies have reported that the characteristics of gut microbiome and metabolites in patients with GDM are significantly altered; however, the specific microbial taxa and metabolic features identified vary across studies and, in some cases, show opposing trends. Increasing evidence suggests that variations in gut microbiota and metabolic profiles can emerge early in pregnancy due to intrinsic host metabolic and inflammatory states rather than dietary differences alone. Such early biological heterogeneity may predispose women to altered glucose regulation, providing a plausible explanation for why first-trimester microbiota–metabolite signatures are detectable before the clinical onset of GDM. However, most existing evidence is derived from observational studies, and the biological mechanisms underlying the association between gut microbiome alterations and the development of GDM remain incompletely understood. The inconsistencies reported across studies are likely attributable to methodological heterogeneity, including differences in study populations, sample sizes, and study designs [35,36]. Moreover, the analysis of gut microbiota or metabolites alone cannot determine which gut microbiota play a key role in the occurrence of GDM, because the gut microbiota and the host are involved in co-metabolic interactions across multiple metabolic pathways. Therefore, conducting multi-omics studies on microbial metabolites, gut microbiota, and host clinical characteristics simultaneously may provide insights into the mechanisms of disease development. However, multi-omics studies that integrate metagenomics and metabolomics in GDM remain limited and are based on cross-sectional designs [31].

Previous studies have analyzed the differences in gut microbiota composition between GDM cases and controls [37,38]. However, these analyses were primarily based on 16S rRNA sequencing, which limited in-depth exploration at the species level and associations with metabolic profiles. Using metagenomics and untargeted Ultra-high Performance LC-MS analysis, we conducted a nested case–control study to examine associations between gut microbiota, serum metabolites in early pregnancy, and subsequent GDM. By jointly characterizing microbial taxa, circulating metabolites, and clinical indicators, this study aims to provide a more comprehensive descriptive view of biological patterns associated with GDM susceptibility.

## 2. Materials and Methods

### 2.1. Study Design and Participants

A nested case–control study was conducted within a cohort established at the Maternal and Child Health Hospital of Hunan Province, China (EC201624). The study was registered at the Chinese Clinical Trial Registry (ChiCTR; registration number ChiCTR1900020652). All participants provided written informed consent before completing an enrollment questionnaire as well as providing biological samples. The detailed methodology of this cohort has been comprehensively described in a previous publication [37]. All pregnant women underwent an OGTT between 24 and 28 weeks of gestation; GDM cases were defined as women whose blood glucose levels at fasting, 1 h, and 2 h after glucose ingestion were ≥5.1 mmol/L, ≥10.0 mmol/L, and ≥8.5 mmol/L, respectively.

Cases were selected according to the following criteria:

(1) GDM was diagnosed at 24–28 weeks of gestation;
(2) Completed questionnaire data and available blood and fecal samples;
(3) No probiotics and antibiotics during pregnancy; and
(4) No history of hypertension, thyroid disease, cardiovascular and cerebrovascular disorders during pregnancy.

Based on age (± 3 years) and gestational age (±1 week) at data collection and sample collection, controls with normal blood glucose throughout pregnancy were randomly selected for each case in a 1:2 ratio.

Participants were excluded if they met any of the following conditions:

(1) Multiple pregnancies;
(2) Inability to cooperate with the investigation due to mental illness or extreme emotional instability;
(3) A child with any congenital disease.

### 2.2. Sample Size Calculation

This study involved 47 case and 94 controls, fulfilling the research requirements. Based on our previous results [37], the gut microbial α-diversity (Simpson index) in women with GDM was 0.945, whereas in women without GDM it was 0.965, with a standard deviation of approximately 0.03. Sample size calculations were performed using PASS (Power Analysis and Sample Size) software (version 25.0.3; NCSS, LLC, Kaysville, UT, USA). With two groups of 47 participants each, a two-sided equal-variance t-test at a significance level of α = 0.05 provides a statistical power of 90.4% to detect a mean difference of 0.02, assuming a common standard deviation of 0.03.

### 2.3. Data Collection

Basic information, including socio-demographic characteristics (age, ethnicity, educational level, employment, number of pregnancies, monthly household income, and health insurance status), lifestyles, disease history and health conditions, medication use, dietary intake, and supplement use were collected through a self-administered questionnaire completed by each participant at enrollment.

3–5 mL of EDTA-anticoagulated peripheral blood samples were collected from all study subjects by a nurse at enrollment. The blood samples were centrifuged at 4 °C for 15 min (4 °C, 3500× *g* rpm) within 12 h; the serum and blood cells were stored at −80 °C until processing. All pregnant women underwent an OGTT between 24 and 28 weeks of gestation. The laboratory test results and uncertain medication information were further confirmed through their Maternal and Child Health Manual and medical records.

### 2.4. Stool Sample Collection and Metagenomic Sequencing

Each woman was given a fecal sampler and provided detailed instructions for sample collection at enrollment. About 10 g fresh fecal samples were collected from each woman and placed immediately at −20 °C. All samples were transported to the laboratory within 12 h.

Fecal bacterial DNA was extracted using QIAamp Fast DNA Mini Kit (QIAGEN, Hilden, Germany) and stored at −80 °C. PCR amplification and sequencing were conducted using the Illumina platform. The amplified PCR products were purified. The purity of DNA from fecal samples was detected using an ultra-micro UV spectrophotometer (Thermo Fisher Scientific, Waltham, MA, USA), adhering to the principle that the DNA detection concentration should be higher than 10 μg/μL, with an optimal optical density (OD) value ranging between 1.8 and 2.0.

1 µg of high-quality DNA was processed to construct sequencing libraries, using the NEBNext^®^ Ultra™ DNA Library Prep Kit for Illumina (New England Biolabs, Ipswich, MA, USA) following the manufacturer’s instructions. Library quality and quantity were assessed using the Agilent 2100 Bioanalyzer (Agilent Technologies, Santa Clara, CA, USA) with a high-sensitivity and wide dynamic range (0.1–50 ng/µL) to ensure that the libraries met Illumina platform quality standards. Absolute quantification of the library was performed using real-time quantitative PCR (qPCR, TaqMan probe method). Cluster generation on the flow cell was conducted using the Illumina cBot system (bridge PCR amplification), with paired-end sequencing libraries constructed using the TruSeq Nano DNA Library Prep Kit (Illumina, San Diego, CA, USA). High-throughput sequencing was ultimately performed on the Illumina HiSeq X Ten platform (2 × 150 bp read length mode), which achieves a single-run throughput of 1.6–1.8 Tb with ≥75% Q30 bases. The sequencing service was completed by Novogene Co., Ltd. (Beijing Novogene Bioinformatics Technology Co., Ltd., Beijing, China).

Raw sequencing reads underwent quality control using KneadData, including adapter removal and removal of host DNA, to generate high-quality clean reads. Taxonomic profiling was performed using MetaPhlAn2 through alignment to microbial reference genomes—including bacteria, archaea, eukaryotes, and viruses—for taxonomic composition and relative abundance annotation. Functional profiling was conducted with HUMAnN3 (v3.0), which mapped reads to pan-genomes and performed translated searches for unclassified reads, ultimately quantifying gene families and metabolic pathways.

### 2.5. Untargeted Metabolomic Assays

After thawing at −80 °C, serum samples underwent the following pretreatment procedures:

(1) Addition of L-2-chlorophenylalanine internal standard solution (prepared in methanol) for quantification;
(2) Protein precipitation using a methanol–acetonitrile mixture (2:1, *v*/*v*);
(3) Acquisition of supernatant after ultrasonication, low-temperature incubation, and subsequent centrifugation;
(4) Preparation of quality control (QC) samples in parallel to assess system stability.

For the liquid chromatography–mass spectrometry (LC–MS) analysis, a UPLC–QTOF–MS/MS system equipped with an Acquity RSL C120 C18 column (1.8 µm, 2.1 × 100 mm) was used. Data acquisition was performed in both ESI+ and ESI− modes. QC samples were used to monitor system stability, equilibrate the LC–MS system, and evaluate data quality for metabolite identification. The UPLC conditions included a C120 C18 column (100 × 2.1 mm), a column temperature maintained at 30 °C, and an injection volume of 5 µL.

The LC parameters for the positive ion mode were as follows: mobile phase A was 0.1% formic acid in water; mobile phase B was 0.1% formic acid in acetonitrile. The LC parameters for the negative ion mode were as follows: mobile phase A was 0.05% ammonia solution; mobile phase B was 0.05% formic acid in acetonitrile. Sample injection volume was 5 µL; elution gradient: 2% B for 0.0–2.0 min, increased to 90% B for 2.0–17.5 min, and returned to 2% B for 17.5–20.0 min, with a flow rate of 0.2 mL/min.

The MS analysis was performed under the following conditions: electrospray ionization (ESI) was used as the ion source, operating in dual ESI+ and ESI− detection modes. High-purity nitrogen served as the auxiliary gas for both spray ionization and desolvation processes. The instrument was set to full-scan mode covering a mass range of 20–1000 m/z, with the drying gas maintained at 200 °C and flowing at 0.2 mL/min.

### 2.6. Statistical Analysis

#### 2.6.1. Metagenomic Sequencing Analysis

Alpha diversity was assessed using the Simpson and Shannon indices, with between-group comparisons conducted through Wilcoxon rank-sum tests. Beta diversity was quantified using Bray–Curtis dissimilarity (abundance-based) and Jaccard distance (presence/absence-based) metrics. Principal coordinate analysis (PCoA) was performed using the ade4 package (v1.7-22) in R (v4.4.1) to visualize sample distribution patterns, while hierarchical clustering via the unweighted pair group method with arithmetic mean (UPGMA) was implemented with the phangorn package (v2.11.1). All visualizations were generated using ggplot2 (v3.5.2) with consistent aesthetic parameters.

LEfSe analysis (LDA score > 4) was performed on the Galaxy platform to identify differential species between groups. The results were presented as an LEfSe (v1.0) clustering tree, LDA score plot, and feature table. Spearman correlation analysis was further conducted to evaluate associations among the identified differential species.

Functional annotation was carried out using the HUMAnN2 (v2.8.1) pipeline. Gene families were first annotated based on the UniRef90 database and then mapped to metabolic pathways via the MetaCyc(v27.0) database. The MinPath (v1.4) algorithm was subsequently applied to identify minimal pathway sets, generating functional pathway abundance profiles that comprehensively reflect the metabolic characteristics of microbial communities.

Functional distribution patterns across different groups were visualized using heatmaps generated with the pheatmap package (v1.0.12) in *R* (v4.4.1). LEfSe (v1.0) analysis (maintaining a consistent LDA score > 4) was implemented on functional gene data to identify group-specific differential metabolic pathways. The Benjamini–Hochberg false discovery rate (FDR) method was employed in differential abundance analysis of species and genera, functional gene and pathway analyses, and intergroup comparisons of KEGG and pathway abundances.

#### 2.6.2. Non-Targeted Metabolomics Data Analysis

Raw data were imported into MetaboScape (version 3.0) for peak picking, noise filtering, and normalization procedures. Subsequent metabolite identification, including metabolite names, molecular formulas, retention times (RT), mass-to-charge ratios (m/z), and peak area values, was reported.

A supervised Orthogonal Partial Least Squares-Discriminant Analysis (OPLS-DA) model was applied as an exploratory multivariate approach to examine the overall metabolic patterns between groups using the ropls package (v1.30.0) in *R* (v4.4.1), and model robustness was assessed through 200 permutation tests to evaluate potential overfitting, with variable importance in projection (VIP) scores calculated as a supportive measure to rank metabolites contributing to group discrimination. Welch’s t-tests were conducted for between-group metabolite comparisons, followed by FDR adjustments. Differentially expressed metabolites were identified based on three criteria: |log_2_(fold change)| > 0.415 (equivalent to >1.33-fold change), BH-adjusted *p*-value < 0.05, and VIP score ≥ 1.

The identified differentially expressed metabolites were subjected to metabolic pathway enrichment analysis using the Kyoto Encyclopedia of Genes and Genomes (KEGG) database (http://www.genome.jp/kegg/; accessed on 25 June 2024). Pathway significance was assessed via Fisher’s exact test (BH-adjusted *p* < 0.05). All statistical results were visualized using the *R* (version 4.4.1) programming environment, including volcano plots displaying metabolite change magnitude (fold change), statistical significance (–log_10_-transformed *p*-values), and VIP scores of significant metabolites.

To identify more potential correlation, a power-oriented statistical strategy was adopted, which means that spearman rank correlation analysis was conducted to examine the associations among differentially abundant microbiota, differentially expressed metabolites, and clinical indicators. An FDR-adjusted *p*-value < 0.05 was considered statistically significant.

## 3. Results

### 3.1. Characteristics of the Study Population

From 2018 onward, we enrolled 636 pregnant women from Hunan Maternal and Child Health Care Hospital who had provided demographic and socio-economic data, serum and fecal samples in the first trimester, and a completed oral glucose tolerance test (OGTT) during the second trimester. Based on the 2011 International Association of Diabetes and Pregnancy Study Groups (IADPSG) criteria, 94 women (15.41%) were diagnosed with GDM. 47 patients with GDM and 94 matched controls were randomly selected. The main characteristics in the first trimester, including lifestyle factors and clinical indicators of the 141 subjects, are summarized in Table 1 (the corresponding information for the 636 women is summarized in Supplementary Table S1). Age, height, body mass index (BMI), fasting glucose, and triglycerides were significantly different between GDM pregnant women and women without GDM, whereas characteristics such as ethnicity, education level, blood pressure, and total cholesterol were comparable between the two groups (*p* > 0.05). No participants reported smoking, but 21.4–23.6% of the women reported passive smoking. Only a small percentage of participants reported drinking, and the proportions of passive smoking and drinking did not differ between GDM pregnant women and women without GDM (*p* > 0.05). In particular, total energy intake and most food-group intakes did not differ significantly between the two groups.

### 3.2. Metagenomic Analysis of Gut Microbes

A total of 328 GB of raw sequencing data was generated, with an average sequencing depth of 7327.21 Mb per sample. The average data validity rate reached 99.73%. Through metagenomic species annotation, 424 gut microbiota species, classified into 146 genera, 60 families, 29 orders, 18 classes, and 10 phyla, were identified.

At the phylum level, the relative abundance of the top four dominant taxa in descending order was as follows: *Firmicutes* (68.96%), *Proteobacteria* (19.35%), *Bacteroidetes* (10.39%), and *Actinobacteria* (1.29%). The top four most abundant genera were as follows: *Faecalibacterium* (26.31%), *Bacteroides* (13.67%), *Eubacterium* (2.14%), and *Roseburia* (1.94%). At the species level, the dominant bacterial species were *Ruminococcus bromii* (65.78%) and *Faecalibacterium prausnitzii* (31.88%), while the relative abundance of remaining species each constituted less than 1%.

**Figure 1:**
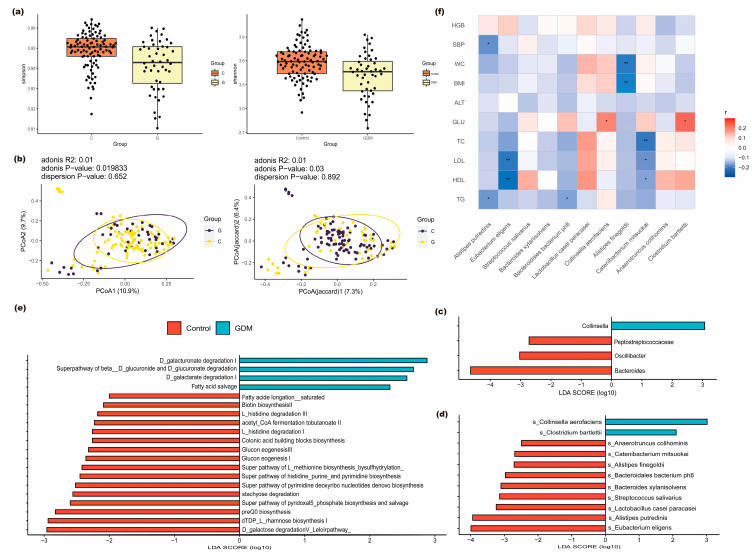
The gut microbial diversity, community composition, and functional differences. (**a**) Compared to non-GDM controls, women with GDM exhibited significantly reduced gut microbial α-diversity, as measured by the Simpson and Shannon indices (*p* < 0.05). (**b**) PCoA based on Bray–Curtis dissimilarity and Jaccard distance metrics further showed distinct clustering patterns between the GDM and control groups. (**c**) Differentially abundant genera between the GDM and control groups identified by LEfSe analysis (BH-adjusted *p* < 0.05, LDA score > 4). (**d**) Differentially abundant species between the GDM and control groups identified by LEfSe analysis (BH-adjusted *p* < 0.05, LDA score > 4). (**e**) Metabolic pathways showing statistically significant differences in abundance between the GDM and control groups. (**f**) Correlations between differential microbial species and clinical indicator. * *p* < 0.05; ** *p* < 0.01.

Compared to non-GDM controls, women with GDM showed significantly reduced gut microbial α-diversity, as measured by both the Simpson index (*p* < 0.05) and Shannon index (*p* < 0.05) (Figure 1a). PCoA based on Bray–Curtis and Jaccard distance metrics showed distinct clustering between the GDM and control groups, suggesting differences in overall community composition (Figure 1b).

Differential analysis of microbial community abundance at both genus and species levels was performed using *LEfSe*. At the genus level, four taxa showed significant differences between the case and control groups. Specifically, the genus *Collinsella* was enriched in the case group, while *Peptostreptococcaceae*, *Oscillibacter*, *and Bacteroides* were significantly more abundant in the control group (as shown in Figure 1c). At the species level, a total of 11 differentially abundant taxa were identified between the two groups. *Collinsella aerofaciens* and *Clostridium bartlettii* were enriched in the case group. In contrast, *Anaerotruncus colihominis*, *Catenibacterium mitsuokai*, *Alistipes finegoldii*, *Bacteroidales bacterium ph8*, *Bacteroides xylanisolvens*, *Streptococcus salivarius*, *Lactobacillus casei paracasei*, *Alistipes putredinis*, and *Eubacterium eligens* showed higher abundance in the control group (as shown in Figure 1d). The corresponding characteristic values and statistical test results are provided in Supplementary Table S2.

Functional annotation of the sequencing data was performed using *HUMAnN2* and successfully identified 401 functional pathways. Subsequent *LEfSe* analysis identified 20 metabolic pathways exhibiting statistically significant differential abundance between the case and control groups, as shown in Figure 1e. Among them, four pathways were significantly enriched in the case group: *D-galactarate degradation I*, *Superpathway of beta-D-glucuronide*, *D-glucuronate degradation*, and *D-galacturonate degradation and Fatty acid salvage* (Supplementary Table S3).

Analysis of the correlation between 11 differentially abundant species at the species level and clinical indicators showed that *Collinsella aerofaciens* positively associated with first-trimester fasting glucose levels (r = 0.19, *p* = 0.04), as did *Clostridium bartlettii* (r = 0.22, *p* = 0.01). The correlation results between other differential bacterial species and additional clinical parameters are presented in Figure 1f.

### 3.3. Serum Metabolomic Profiles and Correlations

**Figure 2:**
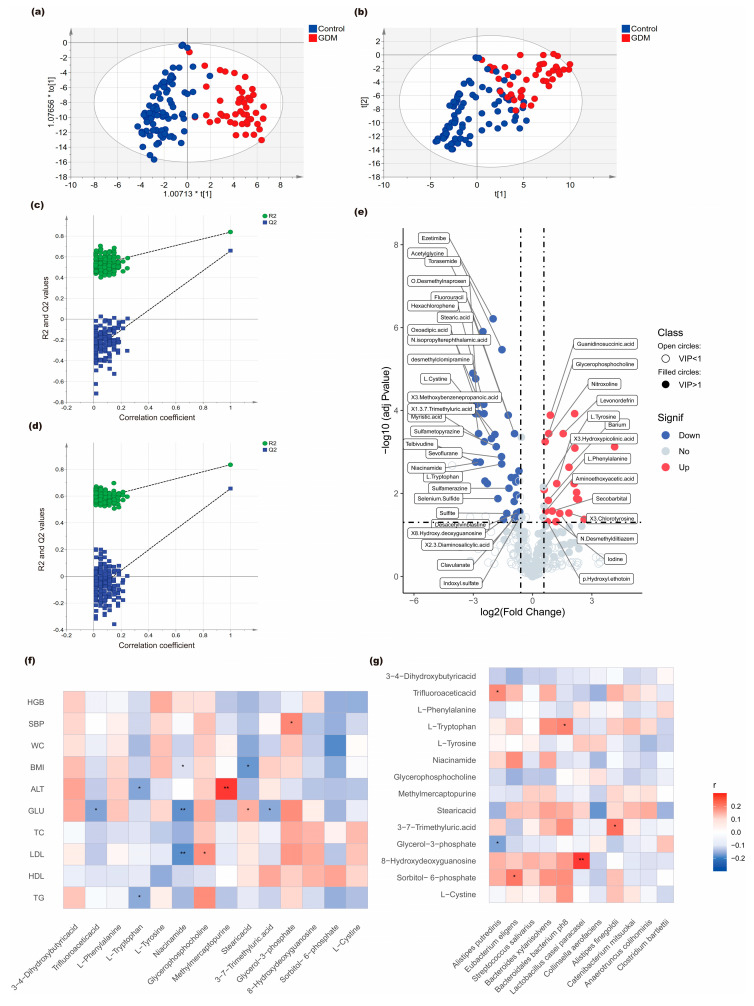
Serum metabolomic alterations and correlation networks among metabolites, gut microbiota, and clinical indicators. (**a**,**b**) OPLS-DA score plots showed group-level separation between the GDM and control groups under positive and negative ion modes, respectively. (**c**,**d**) Permutation tests (200 iterations) assessing the stability of the OPLS-DA models in positive and negative ion modes. (**e**) Volcano plot showed serum metabolites with differential abundance between GDM and control groups (BH-adjusted *p* < 0.05, |log_2_FC| > 0.415, VIP ≥ 1). The vertical dashed lines indicate the fold-change threshold, and the horizontal dashed line indicates the significance threshold. (**f**) Correlations between differentially abundant serum metabolites and clinical indicators. (**g**) Correlations between differentially abundant gut microbial species and serum metabolites. * *p* < 0.05; ** *p* < 0.01.

OA total of 381 metabolites were identified in both case and control groups by untargeted metabolomic analysis via UPLC-MS. OPLS-DA showed an apparent separation of metabolic patterns between GDM cases and controls (Figure 2a,b). The stability of the OPLS-DA model was assessed through 200 permutation tests, suggesting acceptable model performance (Figure 2c,d).

We identified 64 significantly differential metabolites between women with GDM and non-GDM controls using the following stringent criteria: (1) absolute log2 fold change > 0.415, (2) Benjamini–Hochberg adjusted *p*-value < 0.05, and (3) variable importance in projection (VIP) score ≥ 1. The detailed characteristics of the 64 metabolites are provided in Supplementary Table S4.

Among these differentially expressed metabolites, 23 metabolites were upregulated in GDM cases (Figure 2e). GDM cases showed elevated levels of Glycerol-3-phosphate, Glycerophosphocholine, Phenylalanine, Tyrosine, and 3,4-Dihydroxybutyric acid, while controls had higher levels of Cystine, Tryptophan, Niacinamide, Sorbitol-6-phosphate, Stearic acid, Trifluoroacetic acid, 3,7-Trimethyluric acid, Thioguanine, and 8-Hydroxy-2′-deoxyguanosine, etc.

By calculating the Spearman correlation coefficients, the relationship between 14 significant differential serum metabolites and clinical indicators in the first trimester was examined. The results of the correlation matrix indicated that Stearic acid was positively associated with first-trimester fasting glucose levels (r = 0.18, *p* = 0.04), and conversely, 3,7-Trimethyluric acid (r = −0.18, *p* = 0.04), Niacinamide (r = −0.24, *p* = 0.008), and Trifluoroacetic acid (r = −0.19, *p* = 0.03) were negatively associated with first-trimester fasting glucose levels (Figure 2f).

Correlation analysis between 11 significantly differential microbial taxa in the gut microbiota and 14 significant differential serum metabolites indicated that six differential species at the species level were associated with seven differential metabolites. Among these, *Alistipes putredinis* was positively associated with trifluoroacetic acid (r = 0.18, *p* = 0.04) and negatively associated with glycerol-3-phosphate (r = −0.22, *p* = 0.02). *Collinsella aerofaciens* was negatively related to Stearic acid (r = −0.23, *p* = 0.01) and *Alistipes finegoldii* was positively related to 3–7-trimethyluric acid (r = 0.22, *p* = 0.01). The correlations between microbial taxa and metabolites are presented in Figure 2g.

## 4. Discussion

### 4.1. Gut Microbiota Alterations Associated with GDM

Consistent with previous studies, this study observed significant differences in gut microbiota composition, including reduced alpha diversity and increased beta diversity in GDM women compared with non-GDM controls, through the metagenomic analyses. *Firmicutes*, *Proteobacteria*, and *Bacteroidetes* were the predominant phyla, consistent with our previous findings [37] but with slight variations likely attributable to methodological differences between 16S rRNA sequencing and whole metagenome sequencing.

At the species level, eleven differential gut microbiota were identified (all *p* < 0.05). *Collinsella aerofaciens* and *Clostridium bartlettii* were enriched in the GDM group, while nine taxa, including *Alistipes putredinis* and *Bacteroidales bacterium ph8*, were more abundant in controls. The genus *Collinsella* has been reported to be increased in GDM [11] and T2DM patients [39,40], and associated with inflammatory and metabolic phenotypes in prior observational studies [41]. The enrichment of *Clostridium bartlettii* in the GDM group and its positive correlation with fasting blood glucose levels suggested an association with altered glucose-related metabolic states. Previous work also associated *Clostridium bartlettii* with metformin response in diabetic patients [42], and other *Clostridium* species have been reported in GDM-related studies [11,13,43]. In contrast, Bacteroides was enriched in controls [9,11], and has been reported to be associated with fatty acid oxidation and broader aspects of lipid metabolism and insulin sensitivity [44,45]. Two species—*Bacteroidales bacterium ph8* and *Bacteroides xylanisolvens*—were particularly abundant in the control group. *Bacteroidales bacterium ph8* has been reported to be reduced in women with type 1 diabetes [46] and associated with gut-derived metabolites such as *p*-cresyl sulfate.

Several taxa identified in this study have shown inconsistent associations with glucose-related metabolic traits across previous reports. For example, *Oscillibacter* has been variably associated with glucose-related metabolic traits, with both reduced abundance reported in individuals with impaired glucose tolerance or prediabetes [9,47,48] and enrichment observed in women with GDM [49]. In our cohort, *Oscillibacter* was more abundant in non-GDM controls than in GDM cases, further highlighting the heterogeneous directions of association. Similarly, *Alistipes* spp. were negatively correlated with fasting glucose in our cohort, in line with previous reports of similar associations [22,28,31]. In contrast, other studies have reported an increased abundance of *Alistipes* in T2DM [41,50], suggesting its dual role as both protective and proinflammatory depending on metabolic context [51]. In addition, conflicting patterns have been reported for *Eubacterium* species in diabetes-related studies [39,52,53,54,55,56]. While some reported elevated *Eubacterium* in diabetes [39,52], our findings, consistent with Karlsson et al. [56], suggested enriched *Eubacterium eligens* in controls. *Streptococcus* also displayed higher abundance in controls, in line with Zheng et al. [57]. Taken together, the relationships among gut microbiota at the species level, host metabolism, and GDM are highly heterogeneous, warranting further investigation.

### 4.2. Metabolomic Alterations Associated with GDM

Untargeted metabolomic profiling revealed 64 differential metabolites, primarily involving amino acid and lipid metabolism pathways. Elevated phenylalanine and tyrosine in the GDM group align with previous studies [29]. In contrast, tryptophan levels were higher in controls, in line with earlier observational and mechanistic studies highlighting alterations in amino acid metabolism and redox homeostasis in metabolic disorders [58,59,60,61]. Tryptophan showed a negative correlation with triglyceride levels [62]. However, prior studies have reported inconsistent patterns for tryptophan-related metabolites in GDM, highlighting heterogeneity across study populations.

Among lipid-related metabolites, glycerol-3-phosphate and glycerophosphocholine were elevated in GDM cases. Nicotinamide was increased in controls and showed inverse correlations with glucose and LDL levels, consistent with prior findings in metabolic disease contexts [63,64,65]. Other metabolites, including sorbitol-6-phosphate, stearic acid, and purine intermediates, also showed differed between groups, although their biological interpretation remains uncertain and requires further validation.

### 4.3. Integration of Gut Microbiota and Metabolomic Findings

Our study revealed distinct microbial and metabolomic profiles in women with GDM compared with non-GDM controls through integrated metagenomic and metabolomic analyses. Correlation analyses between gut microbes, serum metabolites, and clinical indicators identified patterns of association across these data layers. *Alistipes putredinis*, enriched in controls, was negatively correlated with glycerol-3-phosphate and fasting glucose. *Bacteroidales bacterium ph8* was positively correlated with tryptophan and inversely correlated with triglyceride levels. Together, these cross-domain associations provide a speculative model linking gut microbes, circulating metabolites, and metabolic traits in early pregnancy.

### 4.4. Future Directions

Our integrated analysis highlights the association patterns between gut microbiota and host metabolism in GDM. Future longitudinal and functional studies should further evaluate these microbial and metabolic signatures over the course of pregnancy and assess their potential causal relevance, beyond simply testing previously proposed speculative pathways. Understanding the temporal dynamics of changes in microbiota and metabolite profiles during pregnancy may help improve understanding of early metabolic alterations related to GDM.

## 5. Limitations

This study has several limitations. The sample size was estimated based on the intergroup differences in microbial diversity; however, this approach primarily addresses community-level variations and does not guarantee sufficient statistical power for detailed species-level comparisons or for detecting nuanced metabolomic correlations. Consequently, the robustness of species-specific conclusions may be limited. Additionally, the limited sample size with a relatively stringent LDA threshold increases the risk of overfitting and false positives in subsequent multiple correlation analyses. Given the observational nature of the study and limited sample size, the findings should be interpreted with appropriate caution.

Dietary intake was not adjusted in the analysis for several reasons: (1) the primary aim of this study was to identify potential correlations between microbiota, metabolites, and clinical indicators. Accordingly, a power-oriented statistical strategy was adopted, aiming to increase the sensitivity and facilitate the detection of correlations with moderate effect sizes. Moreover, baseline dietary intake showed no statistically significant differences between the GDM and control groups, and no significant associations were detected in either univariate or multivariate analyses. (2) The dietary patterns of pregnant women in Hunan were relatively homogeneous during the first trimester due to traditional habits, which may have minimized the dietary impact on gut microbiota composition. (3) Strict inclusion and exclusion criteria were applied, with all participants having no history of GDM and no recent (within one month) use of antibiotics or probiotics, ensuring comparability of gut microbiota-influencing factors between groups.

We also noted that obesity was unequally distributed between the two groups at baseline. Obesity undoubtedly affects metabolism, potentially introducing bias into the results. Based on the complex interactions among obesity, gut microbiota, and metabolism, we included waist circumference as a clinical factor in the correlation analysis to better elucidate the relationship between obesity and specific metabolites.

Although this study provides insufficient evidence regarding the mechanisms by which gut microbes influence the onset of GDM, it still offers an overview of gut microbiota and metabolites related to GDM, providing hypotheses for further mechanistic research.

## 6. Conclusions

Despite these limitations, this study provides valuable insights into the relationship between gut microbiota, serum metabolites, and GDM. Using a nested case–control design within a well-characterized cohort of pregnant women, this study suggested that alterations in gut microbiota during the first trimester are associated with later GDM status.

The multi-omics strategy—particularly the use of metagenomics—enhanced sequencing depth and enabled the identification of differential taxa at the species level. Furthermore, the integration of non-targeted metabolomics allowed for the identification of metabolic pathways potentially influenced by the gut microbiota. Together, these findings provide exploratory insights of the development of GDM and new clues for early prevention and intervention strategies.

## Figures and Tables

**Table 1:** General characteristics of GDM and non-GDM groups in the first trimester.

Variable	GDM Group (*n* = 47)	Non-GDM Group (*n* = 94)	*p*-Value
Age	32.96 ± 4.57	29.61 ± 3.85	<0.001
Ethnicity			0.858
Han	45 (95.74)	92 (97.87)	
Other	2 (4.26)	2 (2.13)	
Education level			0.401
≤High school	8 (17.02)	9 (9.57)	
College	13 (27.66)	25 (26.60)	
≥Undergraduate	26 (55.32)	60 (63.83)	
Occupation			0.651
Worker	17 (36.17)	37 (35.58)	
Technician	17 (36.17)	41 (39.42)	
Farmer	7 (14.89)	10 (9.62)	
Other	6 (12.77)	16 (15.38)	
Monthly household income (CNY)			0.516
<5000	5 (10.60)	7 (7.40)	
5000–10,000	19 (40.40)	50 (53.20)	
10,001–20,000	19 (40.40)	31 (33.00)	
>20,000	4 (8.50)	6 (6.40)	
Height (cm)	158.30 ± 3.57	159.79 ± 4.41	0.033
Weight (kg)	57.05 ± 6.92	55.38 ± 10.33	0.258
BMI (kg/m^2^)	22.78 ± 2.70	21.65 ± 3.64	0.040
Systolic blood pressure (mmHg)	119.43 ± 9.27	115.12 ± 15.94	0.045
Diastolic blood pressure (mmHg)	76.45 ± 8.56	74.77 ± 8.83	0.280
Fasting glucose (mmol/L,)	4.89 ± 0.42	4.63 ± 0.38	<0.001
Triglycerides (mmol/L)	1.75 ± 0.70	1.41 ± 0.64	0.007
Total cholesterol (mmol/L,)	4.56 ± 0.69	4.32 ± 1.47	0.188
HDL-C (mmol/L,)	1.72 ± 0.32	1.80 ± 0.67	0.337
LDL-C (mmol/L)	2.56 ± 0.64	2.33 ± 1.01	0.099
Albumin (g/L)	44.85 ± 2.37	45.57 ± 2.56	0.102
ALT (U/L)	24.65 ± 25.42	18.88 ± 17.37	0.165
AST (U/L)	20.75 ± 11.66	19.15 ± 7.62	0.399
Creatinine (µmol/L,)	43.66 ± 5.32	43.85 ± 6.50	0.854
Uric acid (µmol/L)	211.40 ± 49.23	203.35 ± 48.33	0.360
Urea (mmol/L)	2.56 ± 0.62	2.63 ± 0.64	0.546
Obstetric history			
Gravidity			0.045
1	13 (27.66)	43 (45.74)	
≥2	34 (72.34)	51 (54.26)	
Parity			0.101
0	23 (48.94)	61 (64.89)	
≥1	24 (51.06)	33 (35.11)	
Alcohol use			0.988
Yes	3 (3.10)	17 (3.20)	
No	95 (96.90)	521 (96.80)	
Passive smoking			0.291
Yes	8 (17.02)	25 (26.60)	
No	39 (82.98)	69 (73.40)	
Sleep quality			0.574
Good	9 (19.10)	21 (22.30)	
Fair	28 (59.60)	46 (48.90)	
Poor	9 (19.10)	26 (27.70)	
Bad	1 (2.10)	1 (1.10)	
Energy intake (kcal)	1563.12 ±459.30	1563.86 ± 451.16	0.993
Daily cereal intake (g)	150.0 (120.0–217.5)	150.0 (120.0–221.2)	0.505
Daily tuber intake (g)	14.3 (3.3–25.0)	14.3 (5.4–21.4)	0.743
Daily vegetable intake (g)	200.0 (160.0–300.0)	200.0 (120.0–300.0)	0.646
Daily fruit intake (g)	402.43 ± 144.73	365.14 ± 155.35	0.163
Daily meat intake (g)	30.0 (8.6–60.0)	60.0 (20.0–78.8)	0.051
Daily seafood intake (g)	0.0 (0.0–4.0)	0.0 (0.0–4.0)	0.849
Daily fresh water products intake (g)	8.6 (0.0–17.1)	8.6 (4.0–17.1)	0.723
Daily eggs intake (g)	50.0 (21.4–50.0)	50.0 (21.4–50.0)	0.439
Daily milk intake (g)	71.4 (10.0–171.4)	93.9 (28.6–200.0)	0.442
Daily beans intake (g)	14.3 (5.0–32.1)	15.0 (6.4–32.1)	0.722
Daily nuts intake (g)	38.6 (9.6–75.0)	26.8 (6.1–50.0)	0.311
Daily oil intake (g)	22.5 (21.0–24.0)	22.5 (21.0–24.0)	0.989
Daily salt intake (g)	10.98 ± 1.22	10.97 ± 1.28	0.981

## Data Availability

The data that support the findings of this study are available from the corresponding author upon reasonable request. The data are not publicly available due to privacy restrictions and confidentiality agreements.

## Supplementary Materials

The following supporting information can be downloaded at: https://www.mdpi.com/article/10.3390/nu18030381/s1, Supplementary Table S1: Baseline characteristics of the 636 pregnant women enrolled in the study; Supplementary Table S2: Differentially abundant genera and species between GDM and non-GDM groups identified by *LEfSe* analysis; Supplementary Table S3: Differentially abundant metabolic pathways between GDM and non-GDM groups identified by *LEfSe* analysis; Supplementary Table S4: Differential metabolites identified among comparison groups under the OPLS-DA model.

## Abbreviations

The following abbreviations are used in this manuscript:

GDM	Gestational diabetes mellitus
BMI	Body mass index
LC–MS	Liquid chromatography–mass spectrometry
BACC	Branched-chain amino acids
ESI	Electrospray ionization
UPGMA	Unweighted pair group method with arithmetic mean
LDA	Linear discriminant analysis
OPLS-DA	Orthogonal Partial Least Squares-Discriminant Analysis
KEGG	Kyoto Encyclopedia of Genes and Genomes (KEGG)
OGTT	Oral glucose tolerance test
PCoA	Principal coordinates analysis

## References

[1] Nguyen C.L., Pham N.M., Binns C.W., Duong D.V., Lee A.H. (2018). Prevalence of Gestational Diabetes Mellitus in Eastern and Southeastern Asia: A Systematic Review and Meta-Analysis. J. Diabetes Res..

[2] Malaza N., Masete M., Adam S., Dias S., Nyawo T., Pheiffer C. (2022). A Systematic Review to Compare Adverse Pregnancy Outcomes in Women with Pregestational Diabetes and Gestational Diabetes. Int. J. Environ. Res. Public Health.

[3] Kramer C.K., Campbell S., Retnakaran R. (2019). Gestational Diabetes and the Risk of Cardiovascular Disease in Women: A Systematic Review and Meta-Analysis. Diabetologia.

[4] Tobias D.K., Hu F.B., Forman J.P., Chavarro J., Zhang C. (2011). Increased Risk of Hypertension after Gestational Diabetes Mellitus: Findings from a Large Prospective Cohort Study. Diabetes Care.

[5] Medici Dualib P., Ogassavara J., Mattar R., Mariko Koga da Silva E., Atala Dib S., de Almeida Pititto B. (2021). Gut Microbiota and Gestational Diabetes Mellitus: A Systematic Review. Diabetes Res. Clin. Pract..

[6] Hasain Z., Mokhtar N.M., Kamaruddin N.A., Mohamed Ismail N.A., Razalli N.H., Gnanou J.V., Raja Ali R.A. (2020). Gut Microbiota and Gestational Diabetes Mellitus: A Review of Host-Gut Microbiota Interactions and Their Therapeutic Potential. Front. Cell. Infect. Microbiol..

[7] Wang J., Zheng J., Shi W., Du N., Xu X., Zhang Y., Ji P., Zhang F., Jia Z., Wang Y. (2018). Dysbiosis of Maternal and Neonatal Microbiota Associated with Gestational Diabetes Mellitus. Gut.

[8] Wang X., Liu H., Li Y., Huang S., Zhang L., Cao C., Baker P.N., Tong C., Zheng P., Qi H. (2020). Altered Gut Bacterial and Metabolic Signatures and Their Interaction in Gestational Diabetes Mellitus. Gut Microbes.

[9] Crusell M.K.W., Hansen T.H., Nielsen T., Allin K.H., Rühlemann M.C., Damm P., Vestergaard H., Rørbye C., Jørgensen N.R., Christiansen O.B. (2018). Gestational Diabetes Is Associated with Change in the Gut Microbiota Composition in Third Confirmed.Trimester of Pregnancy and Postpartum. Microbiome.

[10] Li G., Yin P., Chu S., Gao W., Cui S., Guo S., Xu Y., Yuan E., Zhu T., You J. (2021). Correlation Analysis between GDM and Gut Microbial Composition in Late Pregnancy. J. Diabetes Res..

[11] Cortez R.V., Taddei C.R., Sparvoli L.G., Ângelo A.G.S., Padilha M., Mattar R., Daher S. (2019). Microbiome and Its Relation to Gestational Diabetes. Endocrine.

[12] Ferrocino I., Ponzo V., Gambino R., Zarovska A., Leone F., Monzeglio C., Goitre I., Rosato R., Romano A., Grassi G. (2018). Changes in the Gut Microbiota Composition during Pregnancy in Patients with Gestational Diabetes Mellitus (GDM). Sci. Rep..

[13] Wei J., Qing Y., Zhou H., Liu J., Qi C., Gao J. (2022). 16S rRNA Gene Amplicon Sequencing of Gut Microbiota in Gestational Diabetes Mellitus and Their Correlation with Disease Risk Factors. J. Endocrinol. Investig..

[14] Chen T., Zhang Y., Zhang Y., Shan C., Zhang Y., Fang K., Xia Y., Shi Z. (2021). Relationships between Gut Microbiota, Plasma Glucose and Gestational Diabetes Mellitus. J. Diabetes Investig..

[15] Zhang X., Wang P., Ma L., Guo R., Zhang Y., Wang P., Zhao J., Liu J. (2021). Differences in the Oral and Intestinal Microbiotas in Pregnant Women Varying in Periodontitis and Gestational Diabetes Mellitus Conditions. J. Oral Microbiol..

[16] Liu H., Pan L.-L., Lv S., Yang Q., Zhang H., Chen W., Lv Z., Sun J. (2019). Alterations of Gut Microbiota and Blood Lipidome in Gestational Diabetes Mellitus with Hyperlipidemia. Front. Physiol..

[17] Hou M. (2020). Changes of Intestinal Flora, Cellular Immune Function and Inflammatory Factors in Chinese Advanced Maternal Age with Gestational Diabetes Mellitus. Acta Medica Mediterr..

[18] Larsen N., Vogensen F.K., van den Berg F.W.J., Nielsen D.S., Andreasen A.S., Pedersen B.K., Al-Soud W.A., Sørensen S.J., Hansen L.H., Jakobsen M. (2010). Gut Microbiota in Human Adults with Type 2 Diabetes Differs from Non-Diabetic Adults. PLoS ONE.

[19] Farhat S., Hemmatabadi M., Ejtahed H.-S., Shirzad N., Larijani B. (2022). Microbiome Alterations in Women with Gestational Diabetes Mellitus and Their Offspring: A Systematic Review. Front. Endocrinol..

[20] Wu N., Zhou J., Mo H., Mu Q., Su H., Li M., Yu Y., Liu A., Zhang Q., Xu J. (2021). The Gut Microbial Signature of Gestational Diabetes Mellitus and the Association with Diet Intervention. Front. Cell. Infect. Microbiol..

[21] Crusell M.K.W., Hansen T., Nielsen T., Allin K., Ruehlemann M., Damm P., Vestergaard H., Roerbye C., Joergensen N., Christiansen O.B. (2018). Gestational Diabetes Is Associated with an Aberrant Gut Microbiota during Pregnancy and Postpartum. J. Reprod. Immunol..

[22] Festa C., Drago L., Martorelli M., Di Marino V.P., Bitterman O., Corleto C.C., Corleto V.D., Napoli A. (2020). Flash on Gut Microbiome in Gestational Diabetes: A Pilot Study. New Microbiol..

[23] Wu Y., Bible P.W., Long S., Ming W.-K., Ding W., Long Y., Wen X., Li X., Deng X., Deng Y. (2020). Metagenomic Analysis Reveals Gestational Diabetes Mellitus-Related Microbial Regulators of Glucose Tolerance. Acta Diabetol..

[24] Kuang Y.-S., Lu J.-H., Li S.-H., Li J.-H., Yuan M.-Y., He J.-R., Chen N.-N., Xiao W.-Q., Shen S.-Y., Qiu L. (2017). Connections between the Human Gut Microbiome and Gestational Diabetes Mellitus. GigaScience.

[25] Su Y., Wang H.-K., Gan X.-P., Chen L., Cao Y.-N., Cheng D.-C., Zhang D.-Y., Liu W.-Y., Li F.-F., Xu X.-M. (2021). Alterations of Gut Microbiota in Gestational Diabetes Patients during the Second Trimester of Pregnancy in the Shanghai Han Population. J. Transl. Med..

[26] Chen F., Gan Y., Li Y., He W., Wu W., Wang K., Li Q. (2021). Association of Gestational Diabetes Mellitus with Changes in Gut Microbiota Composition at the Species Level. BMC Microbiol..

[27] Ye G., Zhang L., Wang M., Chen Y., Gu S., Wang K., Leng J., Gu Y., Xie X. (2019). The Gut Microbiota in Women Suffering from Gestational Diabetes Mellitus with the Failure of Glycemic Control by Lifestyle Modification. J. Diabetes Res..

[28] Hu P., Chen X., Chu X., Fan M., Ye Y., Wang Y., Han M., Yang X., Yuan J., Zha L. (2021). Association of Gut Microbiota during Early Pregnancy with Risk of Incident Gestational Diabetes Mellitus. J. Clin. Endocrinol. Metab..

[29] Sun Z., Pan X.-F., Li X., Jiang L., Hu P., Wang Y., Ye Y., Wu P., Zhao B., Xu J. (2023). The Gut Microbiome Dynamically Associates with Host Glucose Metabolism throughout Pregnancy: Longitudinal Findings from a Matched Case-Control Study of Gestational Diabetes Mellitus. Adv. Sci..

[30] Zhou J., Yu J., Ren J., Ren Y., Zeng Y., Wu Y., Zhang Q., Xiao X. (2025). Association of Maternal Blood Metabolomics and Gestational Diabetes Mellitus Risk: A Systematic Review and Meta-Analysis. Rev. Endocr. Metab. Disord..

[31] Ye D., Huang J., Wu J., Xie K., Gao X., Yan K., Zhang P., Tao Y., Li Y., Zang S. (2023). Integrative Metagenomic and Metabolomic Analyses Reveal Gut Microbiota-Derived Multiple Hits Connected to Development of Gestational Diabetes Mellitus in Humans. Gut Microbes.

[32] Li N., Li J., Wang H., Liu J., Li W., Yang K., Huo X., Leng J., Yu Z., Hu G. (2023). Aromatic Amino Acids and Their Interactions with Gut Microbiota-Related Metabolites for Risk of Gestational Diabetes: A Prospective Nested Case-Control Study in a Chinese Cohort. Ann. Nutr. Metab..

[33] Yu J., Ren J., Ren Y., Wu Y., Zeng Y., Zhang Q., Xiao X. (2024). Using Metabolomics and Proteomics to Identify the Potential Urine Biomarkers for Prediction and Diagnosis of Gestational Diabetes. Ebiomedicine.

[34] Mei S., Chen Y., Long Y., Cen X., Zhao X., Zhang X., Ye J., Gao X., Zhu C. (2025). Association of Gut Microbiota with Overweight/Obesity Combined with Gestational Diabetes Mellitus. J. Med. Microbiol..

[35] Lu W., Hu C. (2022). Molecular Biomarkers for Gestational Diabetes Mellitus and Postpartum Diabetes. Chin. Med. J..

[36] Heath H., Rosario R., McMichael L.E., Fanter R., Alarcon N., Quintana-Diaz A., Pilolla K., Schaffner A., Jelalian E., Wing R.R. (2023). Gestational Diabetes Is Characterized by Decreased Medium-Chain Acylcarnitines and Elevated Purine Degradation Metabolites across Pregnancy: A Case-Control Time-Course Analysis. J. Proteome Res..

[37] Ma S., You Y., Huang L., Long S., Zhang J., Guo C., Zhang N., Wu X., Xiao Y., Tan H. (2020). Alterations in Gut Microbiota of Gestational Diabetes Patients during the First Trimester of Pregnancy. Front. Cell. Infect. Microbiol..

[38] Wu X., Liu X., Tan H., Song J., Ma S., Tan Y. (2025). Longitudinal Change and Causal Relationship between Gut Microbiota and Gestational Diabetes Mellitus. Diabetol. Metab. Syndr..

[39] Zhang X., Shen D., Fang Z., Jie Z., Qiu X., Zhang C., Chen Y., Ji L. (2013). Human Gut Microbiota Changes Reveal the Progression of Glucose Intolerance. PLoS ONE.

[40] Zhong H., Ren H., Lu Y., Fang C., Hou G., Yang Z., Chen B., Yang F., Zhao Y., Shi Z. (2019). Distinct Gut Metagenomics and Metaproteomics Signatures in Prediabetics and Treatment-Naïve Type 2 Diabetics. Ebiomedicine.

[41] Qin J., Li Y., Cai Z., Li S., Zhu J., Zhang F., Liang S., Zhang W., Guan Y., Shen D. (2012). A Metagenome-Wide Association Study of Gut Microbiota in Type 2 Diabetes. Nature.

[42] Elbere I., Silamikelis I., Dindune I.I., Kalnina I., Briviba M., Zaharenko L., Silamikele L., Rovite V., Gudra D., Konrade I. (2020). Baseline Gut Microbiome Composition Predicts Metformin Therapy Short-Term Efficacy in Newly Diagnosed Type 2 Diabetes Patients. PLoS ONE.

[43] Allegretti J.R., Kassam Z., Osman M., Budree S., Fischer M., Kelly C.R. (2018). The 5D Framework: A Clinical Primer for Fecal Microbiota Transplantation to Treat Clostridium Difficile Infection. Gastrointest. Endosc..

[44] Syme C., Czajkowski S., Shin J., Abrahamowicz M., Leonard G., Perron M., Richer L., Veillette S., Gaudet D., Strug L. (2016). Glycerophosphocholine Metabolites and Cardiovascular Disease Risk Factors in Adolescents: A Cohort Study. Circulation.

[45] Würtz P., Soininen P., Kangas A.J., Rönnemaa T., Lehtimäki T., Kähönen M., Viikari J.S., Raitakari O.T., Ala-Korpela M. (2013). Branched-Chain and Aromatic Amino Acids Are Predictors of Insulin Resistance in Young Adults. Diabetes Care.

[46] Roth-Schulze A.J., Penno M.A.S., Ngui K.M., Oakey H., Bandala-Sanchez E., Smith A.D., Allnutt T.R., Thomson R.L., Vuillermin P.J., Craig M.E. (2021). Type 1 Diabetes in Pregnancy Is Associated with Distinct Changes in the Composition and Function of the Gut Microbiome. Microbiome.

[47] Liang Y.-Y., Liu L.-Y., Jia Y., Li Y., Cai J.-N., Shu Y., Tan J.-Y., Chen P.-Y., Li H.-W., Cai H.-H. (2022). Correlation between Gut Microbiota and Glucagon-like Peptide-1 in Patients with Gestational Diabetes Mellitus. World J. Diabetes.

[48] Wu H., Tremaroli V., Schmidt C., Lundqvist A., Olsson L.M., Krämer M., Gummesson A., Perkins R., Bergström G., Bäckhed F. (2020). The Gut Microbiota in Prediabetes and Diabetes: A Population-Based Cross-Sectional Study. Cell Metab..

[49] Dreisbach C., Prescott S., Alhusen J., Dudley D., Trinchieri G., Siega-Riz A.M. (2022). Association between Microbial Composition, Diversity, and Function of the Maternal Gastrointestinal Microbiome with Impaired Glucose Tolerance on the Glucose Challenge Test. PLoS ONE.

[50] Wu X., Ma C., Han L., Nawaz M., Gao F., Zhang X., Yu P., Zhao C., Li L., Zhou A. (2010). Molecular Characterisation of the Faecal Microbiota in Patients with Type II Diabetes. Curr. Microbiol..

[51] Parker B.J., Wearsch P.A., Veloo A.C.M., Rodriguez-Palacios A. (2020). The Genus Alistipes: Gut Bacteria with Emerging Implications to Inflammation, Cancer, and Mental Health. Front. Immunol..

[52] Zhao H., Li H., Chung A.C.K., Xiang L., Li X., Zheng Y., Luan H., Zhu L., Liu W., Peng Y. (2019). Large-Scale Longitudinal Metabolomics Study Reveals Different Trimester-Specific Alterations of Metabolites in Relation to Gestational Diabetes Mellitus. J. Proteome Res..

[53] Kamińska K., Stenclik D., Błażejewska W., Bogdański P., Moszak M. (2022). Probiotics in the Prevention and Treatment of Gestational Diabetes Mellitus (GDM): A Review. Nutrients.

[54] Ericson U., Brunkwall L., Hellstrand S., Nilsson P.M., Orho-Melander M. (2020). A Health-Conscious Food Pattern Is Associated with Prediabetes and Gut Microbiota in the Malmö Offspring Study. J. Nutr..

[55] Zhao L., Lou H., Peng Y., Chen S., Zhang Y., Li X. (2019). Comprehensive Relationships between Gut Microbiome and Faecal Metabolome in Individuals with Type 2 Diabetes and Its Complications. Endocrine.

[56] Karlsson F.H., Tremaroli V., Nookaew I., Bergström G., Behre C.J., Fagerberg B., Nielsen J., Bäckhed F. (2013). Gut Metagenome in European Women with Normal, Impaired and Diabetic Glucose Control. Nature.

[57] Zheng W., Xu Q., Huang W., Yan Q., Chen Y., Zhang L., Tian Z., Liu T., Yuan X., Liu C. (2020). Gestational Diabetes Mellitus Is Associated with Reduced Dynamics of Gut Microbiota during the First Half of Pregnancy. Msystems.

[58] Ma T., Cheng H., Li T., Chen Y., Cai T., Bai J., Wu Z., Xia X., Liang T., Du Y. (2022). N-Acetyl-l-Tryptophan Inhibits CCl4-Induced Hepatic Fibrogenesis via Regulating TGF-Β1/SMAD and Hippo/YAP1 Signal. Bioorg. Chem..

[59] Noto A., Fanos V., Barberini L., Grapov D., Fattuoni C., Zaffanello M., Casanova A., Fenu G., De Giacomo A., De Angelis M. (2014). The Urinary Metabolomics Profile of an Italian Autistic Children Population and Their Unaffected Siblings. J. Matern.-Fetal Neonatal Med..

[60] Houtkooper R.H., Cantó C., Wanders R.J., Auwerx J. (2010). The Secret Life of NAD+: An Old Metabolite Controlling New Metabolic Signaling Pathways. Endocr. Rev..

[61] Memisoğullari R., Taysi S., Bakan E., Capoglu I. (2003). Antioxidant Status and Lipid Peroxidation in Type II Diabetes Mellitus. Cell Biochem. Funct..

[62] Zhang P.-P., Li L.-L., Han X., Li Q.-W., Zhang X.-H., Liu J.J., Wang Y. (2020). Fecal Microbiota Transplantation Improves Metabolism and Gut Microbiome Composition in Db/Db Mice. Acta Pharmacol. Sin..

[63] Xiao Y., Wang Q., Zhang H., Nederlof R., Bakker D., Siadari B.A., Wesselink M.W., Preckel B., Weber N.C., Hollmann M.W. (2024). Insulin and Glycolysis Dependency of Cardioprotection by Nicotinamide Riboside. Basic Res. Cardiol..

[64] Liu J.-R., Deng Z.-H., Zhu X.-J., Zeng Y.-R., Guan X.-X., Li J.-H. (2021). Roles of Nicotinamide N-Methyltransferase in Obesity and Type 2 Diabetes. Biomed Res. Int..

[65] Song Q., Zhou X., Xu K., Liu S., Zhu X., Yang J. (2023). The Safety and Antiaging Effects of Nicotinamide Mononucleotide in Human Clinical Trials: An Update. Adv. Nutr..

