# Supplementary material for: Study on Association Between Gut Microbiota, Serum Metabolism and Gestational Diabetes Mellitus Based on Metagenomic and Metabolomics Analysis
Source: Nutrients. 2026 Jan 23;18(3):381. doi: 10.3390/nu18030381 (PMC12899338; doi:10.3390/nu18030381)
Supplement: Supplementary file 1 [file nutrients-18-00381-s001.zip › nutrients-4054211-supplementary.pdf]

Supplementary Table S1. Differentially abundant genera and specises between GDM and non GDM groups identified by *LEfSe* analysis.

Variable	GDM group (n=98)	Non-GDM group (n=538)	*p*-Value
Age	31.28 ± 4.36	29.84 ± 3.92	0.064
Ethnicity			0.772
Han	96 (98.00)	523 (97.20)	
Other	2 (2.00)	15 (2.80)	
Height (cm)	160.85 ± 4.37	160.52 ± 4.32	0.583
Weight (kg)	61.12 ± 7.15	56.33 ± 6.52	<0.001
BMI (kg/m²)	23.56 ± 2.83	21.88 ± 2.69	<0.001
Systolic blood pressure (mmHg)	112.42 ± 9.52	110.63 ± 9.11	0.179
Diastolic blood pressure (mmHg)	71.45 ± 6.92	70.33 ± 6.81	0.242
Fasting glucose (mmol/L,)	4.85 ± 0.42	4.61 ± 0.38	<0.001
Triglycerides (mmol/L)	1.69 ± 0.49	1.54 ± 0.42	0.024
Total cholesterol (mmol/L,)	5.14 ± 0.91	5.02 ± 0.86	0.327
HDL-C (mmol/L,)	1.58 ± 0.27	1.62 ± 0.28	0.214
LDL-C (mmol/L)	2.92 ± 0.69	2.84 ± 0.65	0.412
Albumin (g/L)	43.72 ± 2.85	43.54 ± 2.96	0.642
ALT (U/L)	18.21 ± 7.62	17.68 ± 8.01	0.504
AST (U/L)	21.35 ± 6.93	20.81 ± 7.24	0.568
Creatinine (µmol/L,)	47.21 ± 5.34	46.84 ± 5.26	0.653
Uric acid (µmol/L)	193.42 ± 35.11	185.97 ± 31.44	0.071
Urea (mmol/L)	2.51 ± 0.48	2.49 ± 0.46	0.737
Obstetric history			
Gravidity			0.212
1	43 (43.90)	278 (51.70)	
≥2	55 (56.10)	260 (48.30)	
Parity			0.164
0	66 (67.30)	402 (74.70)	
≥1	32 (32.70)	136 (25.30)	
Education level			0.684
≤High school	41 (41.80)	211 (39.20)	
College	35 (35.70)	183 (34.00)	
≥Undergraduate	22 (22.50)	144 (26.80)	
Occupation			0.892
Worker	26 (26.50)	148 (27.50)	
Technician	22 (22.40)	111 (20.60)	

Farmer	18 (18.40)	93 (17.30)	
Other	32 (32.70)	186 (34.60)	
Monthly household income(CNY)			0.516
<5000	5 (10.60)	7 (7.40)	
5000–10000	19 (40.40)	50 (53.20)	
10001–20000	19 (40.40)	31 (33.00)	
>20000	4 (8.50)	6 (6.40)	
Alcohol use			0.988
Yes	3 (3.10)	17 (3.20)	
No	95 (96.90)	521 (96.80)	
Passive smoking			0.646
Yes	21 (21.40)	127 (23.60)	
No	77 (78.60)	411 (76.40)	
Sleep quality			0.574
Good	9 (19.10)	21 (22.30)	
Fair	28 (59.60)	46 (48.90)	
Poor	9 (19.10)	26 (27.70)	
Bad	1 (2.10)	1 (1.10)	

Note: Continuous variables are presented as mean ± standard deviation and categorical variables as number (percentage). p-values were calculated using t-tests or chi-square tests, as appropriate. GDM refers to gestational diabetes mellitus.

Supplementary Table S2. Differentially abundant genera and **specises** between GDM and non GDM groups identified by *LEfSe* analysis.

	Maximum Mean Abundance (log_{10})	LDA Score	*p*-Value	Enriched group
**Genera**				
Collinsella	3.64	3.07	0.040	GDM group
Peptostreptococcaceae	3.25	2.72	0.020	Control group
Oscillibacter	3.81	3.03	0.040	Control group
Bacteroides	5.36	4.65	0.002	Control group
**Specises**				
Collinsella aerofaciens	3.58	3.02	0.006	GDM group
Clostridium bartlettii	3.64	2.10	0.040	GDM group
Anaerotruncus colihominis	2.93	2.49	0.005	Control group
Catenibacterium mitsuokai	2.99	2.69	0.030	Control group
Alistipes finegoldii	3.31	2.71	0.030	Control group
Bacteroidales bacterium ph8	3.55	2.97	0.030	Control group
Bacteroides xylanisolvens	3.64	3.10	0.050	Control group
Streptococcus salivarius	3.74	3.14	0.040	Control group
Lactobacillus casei paracasei	3.51	3.24	0.030	Control group
Alistipes putredinis	4.57	3.94	0.030	Control group
Eubacterium eligens	4.57	3.99	0.005	Control group

Note: Differentially abundant genera and species were identified using LEfSe analysis. The significance threshold was set at $p < 0.05$ and LDA score > 2.0. “Enriched group” indicates the group in which the taxon was relatively more abundant. GDM, gestational diabetes mellitus; LDA, linear discriminant analysis.

Supplementary Table S3. Differentially abundant metabolic pathways between GDM and non GDM groups identified by *LEfSe* analysis.

Metabolic pathways	Maximum Mean Abundance (log_{10})	LDA Score	p-Value	Enriched/ Depleted in GDM
D_galactarate degradation I	2.99	2.57	0.040	enriched
D_galacturonate degradation I	3.72	2.88	＜0.001	enriched
Fatty acid salvage	2.6	2.31	＜0.001	enriched
Superpathway of beta__D_glucuronide and D_glucuronate degradation	3.65	2.67	0.040	enriched
acetyl_CoA fermentation tobutanoate II	3.02	2.23	0.001	depleted
Biotin biosynthesisII	2.89	2.09	0.030	depleted
Colonic acid building blocks biosynthesis	3.04	2.26	0.001	depleted
D_galactose degradationV_Leloirpathway_	3.89	2.96	0.020	depleted
dTDP_L_rhamnose biosynthesis I	3.97	2.94	0.040	depleted
Fatty acide longation__saturated	2.84	2.00	＜0.001	depleted
Glucon eogenesis I	3.07	2.36	＜0.001	depleted
Glucon eogenesisIII	3.10	2.32	0.004	depleted
L_histidine degradation I	3.09	2.26	0.003	depleted
L_histidine degradation III	2.98	2.18	0.008	depleted
preQ0 biosynthesis	3.89	2.83	0.020	depleted
stachyose degradation	3.84	2.56	0.030	depleted
Super pathway of histidine_purine_and pyrimidine biosynthesis	3.46	2.45	0.003	depleted
Super pathway of L_methionine biosynthesis_bysulfhydrylation_	3.10	2.42	0.001	depleted
Super pathway of pyridoxal5_phosphate biosynthesis and salvage	3.54	2.60	0.030	depleted
Super pathway of pyrimidine deoxyribo nucleotides denovo biosynthesis	3.44	2.52	0.009	depleted

Note: Differential metabolic pathways were identified using LEfSe with an LDA score > 2.0 and $p < 0.05$. “Enriched” or “Depleted” in GDM indicates whether the pathway abundance was higher or lower in the GDM group compared with controls. GDM, gestational diabetes mellitus; LDA, linear discriminant analysis.

Supplementary Table S4. Differential Metabolites Identified Among Comparison Groups under the OPLS-DA Model

Metabolites	VIP	Mean (GDM)	Variance (GDM)	Mean (CTR)	Variance (CTR)	$log_2(FC)$	Increased/Decreased in GDM	p-Value
Methyluric.acid	2.6	3265.89	4678.34	183.86	263.33	4.151	increased	0.001
CDP	1.5	34979.59	72196.7	4211.96	23127	3.054	increased	0.038
Glycerol.3.phosphate	1.7	8198.58	16301.76	1341.52	6014.68	2.612	increased	0.042
Sulfapyridine	1.76	6691.71	10611.41	1338.03	4908.31	2.322	increased	0.014
Testolactone	1.75	4855.35	7635.66	1032.02	2758.83	2.234	increased	0.013
Levetiracetam	2.02	8609.89	12940.52	1837.22	4620.32	2.228	increased	0.009
S_.3.4.Dihydroxybutyric.acid	2.24	892208.85	1010177.31	202145.43	541431.97	2.142	increased	0.001
Polystyrene.sulfonate	2.33	10725.02	10159.68	2451.23	6284.63	2.129	increased	＜0.001
trans.4.Hydroxycyclohexylacetic.acid	2.04	9675.45	13321.74	2253.78	5581.29	2.102	increased	0.006
Barium	2.03	4473.1	5213.87	1247.34	2526.8	1.842	increased	0.002
Chlorotyrosine	1.6	2887.4	4581.6	807.54	1900.02	1.838	increased	0.03
Levonordefrin	2.14	7150.18	6126.7	2442.23	4632.79	1.55	increased	＜0.001
N.Desmethyldiltiazem	1.36	7768.19	9807.86	2992.92	7511.64	1.376	increased	0.03
Hydroxypicolinic.acid	1.68	8082.19	7330.89	3462.87	6950.41	1.223	increased	0.006
Iodine	1.24	8605.2	11407.6	3788.06	5852.07	1.184	increased	0.048
Secobarbital	1.3	57874.01	56885.15	29014.3	47335.42	0.996	increased	0.027
Guanidinosuccinic.acid	2.15	29390.74	16787.31	15736.7	11765.94	0.901	increased	＜0.001
Glycerophosphocholine	2.41	32976.41	18588.75	18721.16	13223.26	0.817	increased	＜0.001
L.Phenylalanine	1.79	292656.23	243258.36	168838.32	132768.45	0.794	increased	0.015
p.Hydroxyl.ethotoin	1.13	5716.73	5083.22	3421.19	4053.04	0.741	increased	0.048
Aminoethoxy acetic acid	1.45	134173.59	102933.47	84250.76	67723.36	0.671	increased	0.027
Nitroxoline	1.67	14007.97	6229.77	8998.03	6701.6	0.639	increased	0.001
L.Tyrosine	1.39	43517.39	24113.48	29005.09	22178.16	0.585	increased	0.008
Indoxyl.sulfate	1	6308.09	5435.12	9680.35	8606.15	-0.618	decreased	0.036

Metabolites	VIP	Mean (GDM)	Variance (GDM)	Mean (CTR)	Variance (CTR)	log_2(FC)	Increased/ Decreased in GDM	p-Value
Desacetylvinblastine	1.35	545.41	361.23	856.87	887.51	-0.652	decreased	0.028
Gamma.Butyrolactone	1.67	133556.99	120357.4	215256.94	115442.39	-0.689	decreased	0.003
Diaminosalicylic.acid	1.18	3299.66	4406.85	5772.3	5396.35	-0.807	decreased	0.03
Phenylalanylphenylalanine	1.12	75900.33	80701.49	133081.67	122048.75	-0.81	decreased	0.011
Pentosidine	1.42	3128.74	3727.55	5487.07	4423.51	-0.81	decreased	0.011
Methylmercaptopurine	1.29	9945.54	9423.84	17866.48	16753.9	-0.845	decreased	0.005
Desacetylcefotaxime	1.34	418.72	399.97	762.43	706.52	-0.865	decreased	0.004
Clavulanate	1.25	177541.77	313840.01	332926.92	310697.95	-0.907	decreased	0.039
Stearic.acid	1.59	6665.37	7397.12	12559.45	6664.86	-0.914	decreased	＜0.001
Trifluoroacetic.acid	1.53	275405.65	477363.52	524654.29	332237.24	-0.93	decreased	0.016
Pyridoxamine	1.07	363.3	260.06	766.57	986.88	-1.077	decreased	0.004
Sulfamerazine	1.08	1118.38	1346.39	2559.56	3566.76	-1.194	decreased	0.008
Fluorouracil	1.71	45414.94	64739.68	106605.36	81379.01	-1.231	decreased	＜0.001
Sulfite	1.05	2925.15	7288.33	7230.29	10061.67	-1.306	decreased	0.03
Hydroxy.deoxyguanosine	1.22	1397.98	3827.8	3883.3	7001.01	-1.474	decreased	0.043
Torasemide	1.72	277.48	296.26	811.41	769.92	-1.548	decreased	＜0.001
L.Tryptophan	1.51	34797.78	83197.06	102962.59	119341.39	-1.565	decreased	0.002
Sevoflurane	1.48	2719.52	6118.44	8090.48	9395.91	-1.573	decreased	0.001
Selenium.Sulfide	1.33	781.38	2645.01	2638.02	4129.7	-1.755	decreased	0.013
Sulfametopyrazine	1.39	593.54	909.06	2120.16	3214.35	-1.837	decreased	0.001
L.Cystine	1.35	736.83	1872.06	2758.9	3491.36	-1.905	decreased	＜0.001
Ezetimibe	2.11	121	152.3	482.89	502.49	-1.997	decreased	＜0.001
Methoxybenzenepropanoic.acid	1.34	3166.17	3773.39	13409.53	21649.86	-2.082	decreased	＜0.001
Nalidixic.Acid	1.43	328.89	242.51	1607.2	3430.23	-2.289	decreased	0.006
Eplerenone	1.3	2692.34	9127.08	14289.25	28221.53	-2.408	decreased	0.005
Myristic.acid	1.59	49509.34	101104.49	269427.46	465802.76	-2.444	decreased	0.001

Metabolites	VIP	Mean (GDM)	Variance (GDM)	Mean (CTR)	Variance (CTR)	log_2(FC)	Increased/ Decreased in GDM	p-Value
N.isopropylterephthalamic.acid	1.47	312.5	919.54	1712.07	2451.53	-2.454	decreased	＜0.001
Hexachlorophene	1.49	15889.05	50286.5	87966.8	117463.34	-2.469	decreased	＜0.001
Acetylglycine	1.99	216.87	292.06	1236.75	1519.65	-2.512	decreased	＜0.001
Niacinamide	1.57	7674.63	27819.98	48117.75	89869.56	-2.648	decreased	0.002
desmethylclomipramine	1.71	742.76	2189.64	4922.6	7604.8	-2.728	decreased	＜0.001
3.7.Trimethyluric.acid	1.47	872.5	482.49	5788.01	10364.22	-2.73	decreased	＜0.001
Oxoadipic.acid	1.61	3692.41	16181.92	26139.48	35912.75	-2.824	decreased	＜0.001
O.Desmethylnaproxen	1.74	893.83	2714.64	6565.99	9208.52	-2.877	decreased	＜0.001
3._4.Hydroxy.3.methoxyphenyl_.2.methylpropionic.acid	1.55	115.72	162.63	851.1	1410.24	-2.879	decreased	＜0.001
Telbivudine	1.36	211	677.64	1556.84	3110.54	-2.883	decreased	0.002
Hydroxybenzaldehyde	1.73	8973.38	32167.46	72840.4	100693.13	-3.021	decreased	＜0.001
Tamsulosin	2.7	531.49	1194.77	4876.19	4086.42	-3.198	decreased	＜0.001
Sorbitol.6.phosphate	2.43	195.74	221.56	3219.72	3477.9	-4.04	decreased	＜0.001
Hydroxydodecanedioic.acid	1.6	249.02	475.44	8764.15	16760.89	-5.137	decreased	＜0.001

Note:Differential metabolites were identified using orthogonal partial least squares-discriminant analysis (OPLS-DA). Selection criteria were $|log_2(FC)| > 0.415$, VIP ≥ 1.0, and Benjamini–Hochberg adjusted p-value < 0.05. “Increased” or “Decreased” in GDM indicates higher or lower metabolite levels in the GDM group relative to controls. GDM, gestational diabetes mellitus; CTR, control; VIP, variable importance in projection; FC, fold change.
